# Organ-on-a-chip technologies to study neuromuscular disorders: possibilities, limitations, and future hopes

**DOI:** 10.1515/medgen-2021-2085

**Published:** 2021-12-03

**Authors:** Marlen C. Lauffer

**Affiliations:** Institute of Human Genetics, University Hospital Cologne, Kerpener Str. 34, 50931 Cologne, Germany

**Keywords:** disease modeling, induced pluripotent stem cells, neuromuscular disorders, neuromuscular junction, organ-on-a-chip

## Abstract

Neuromuscular disorders are a heterogeneous group of diseases ranging from mild to devastating phenotypes depending on the disorder’s origin. Pathophysiologies for many of these disorders are not fully understood and efficient therapies are urgently needed. Recent advances in the field of induced pluripotent stem cells and organ-on-a-chip technologies have brought enormous improvement in modeling neuromuscular diseases. Even complex units, like the neuromuscular junction, can now be built, enabling researchers to study each component of the motor unit by itself or interacting with others, allowing the identification of disease mechanisms. This article aims to introduce these new modeling systems to study neuromuscular disorders and the possibilities of organ-on-a-chip platforms to shed light on disease pathologies and their use for therapy development.

## Neuromuscular disorders

Neuromuscular disorders (NMDs) comprise a heterogeneous group of disorders that cause dysfunction of the voluntary muscles. Dysfunction originates from defects in the motor unit, whose main components are the skeletal muscle (SkM) fibers and the innervating motoneuron (MN), connected through the neuromuscular junction (NMJ). The function of the motor unit can be disrupted through infections, autoimmune diseases, environmental toxins or due to genetic variations, all of which lead to symptoms of motor impairment and eventually muscular atrophy. Commonly known NMDs include amyotrophic lateral sclerosis, Charcot–Marie–Tooth disease, Duchenne muscular dystrophy (DMD), myasthenia gravis, and spinal muscular atrophy (SMA) [1], [2].

In previous years, advances were made in the development of therapies for hereditary NMDs. In Europe, three approved drugs are available for SMA, and five are available for DMD [3]. Yet, inherited NMDs can still not be cured and the underlying pathomechanisms are often not fully understood. Especially identifying which tissues and cell types are primarily affected and what are secondary effects of the diseases remains challenging. Answering these questions, however, will greatly help to determine new therapeutic targets and improve the current treatment regimen.

To study NMDs, animal models are predominantly used. While these systems have proven very effective, they do have serious limitations that nowadays can partially be overcome by new technologies [4]. Especially when it comes to studying genetic defects that are solely found in humans, for example in genes that have no homologs/orthologs in rodents or play different roles in these species, other modeling systems become necessary. Furthermore, while some hallmarks of human diseases can be captured by animal models, other phenotypes identified in patients cannot [5]. This means disease severity, onset, and progression in animal models may vary widely from humans. Additionally, compounds tested effective in mice many times proved non-efficient in humans [6]. One likely reason for this phenomenon is the heterogeneity of the population, including genetic diversity as well as environmental factors that lead to differences in treatment responses, which currently are not modeled by the highly standardized animal models, such as inbred mouse strains. To account for the heterogeneity and complexity of NMDs and to enable testing of multiple compounds in a high-throughput manner, other modeling systems are required [1]. Furthermore, ethical considerations regarding the reduction and replacement of animal testing (applying the 3R principle [7]) drive the development of alternatives in the field of translational medicine.

New technologies, like miniaturized systems to mimic the human motor unit, can elucidate the pathophysiology of NMDs and advance drug development. With the advent of human induced pluripotent stem cells (hiPSCs) to study human disease, there is now a powerful tool available to investigate a multitude of genetic variants. Cell lines can either be generated directly from patients, or disease-causing mutations are genetically engineered into wild-type stem cell lines, for example via CRISPR [8]. When these hiPSCs are studied in compartmentalized chambers that allow to culture iPSC-derived MNs and SkM separately to form a human motor unit, this is called an organ-on-a-chip system (more precise: NMJ-on-a-chip).

## Organ-on-a-chip technologies

Organs-on-a-chip – synonymously termed organs-on-chip, tissue chips, biochips, or microphysiological systems – encompass a group of small devices used to recapitulate and mimic human organs. Ideally, these bioengineered microdevices are capable of modeling (patho-)physiological responses to environmental cues and stressors as well as natural and synthetic compounds. There is no clear definition of what can be called an organ-on-a-chip; however, common features are the use of multiple cell types in a compartmentalized chamber that includes features that enable physiologically relevant readouts. Another characteristic of organs-on-a-chip is the presence of forces relevant to the tissue/organ being studied, for example, hemodynamic shear forces for vasculature and stretch forces for lung tissue [4]. First steps towards organs-on-a-chip were taken as early as the mid-1990s, with the actual first “lung-on-a-chip” system being published in 2010 [4]. Although organs-on-a-chip were initially developed for the use with primary or established cell lines to model diseases, nowadays the field of iPSC-based miniature devices is expanding rapidly. This expansion is further accelerated by the possibility to grow 3D cultures, advancements in cell culture and differentiation protocols, bio-printing technologies, improved biosensors, microfluidics, and others [4], [9]. Even organoids are grown as organoids-on-a-chip [10].

The design of these platforms is highly dependent on the research question and the desired readouts. Inevitably, one has to decide between (i) bespoke, complex systems with multiple functions and readout possibilities but low throughput and (ii) simpler, high-throughput platforms to screen multiple cell lines and compounds simultaneously. As a consequence, systems are barely standardized and there is a wide variety of designs [4].

Organs-on-a-chip are generally handled as a useful addition and potential replacement of animal models in preclinical research. Key advantages of these platforms include the possibility to tightly control the environment of the tissues being studied, including the cellular architecture and tissue composition as well as biomechanical forces and chemical gradients, and the variety of real-time, functional readouts, like live-cell imaging or electrophysiological measurements. These systems can thus be used to inform about developmental processes and pathophysiologies in a variety of disorders, and their scalability makes them ideally suited for human drug development [4], [9].

## Neuromuscular junctions-on-a-chip

Especially for NMDs, organ-on-a-chip platforms provide the opportunity to take the heterogeneity and complexity of the diseases into account and shed light on their pathophysiologies. With the motor unit being predominantly affected in NMDs, generating NMJs-on-a-chip (NMJ-chips) that incorporate the components of the motor unit is a first step in the right direction [1].

**Figure 1:**
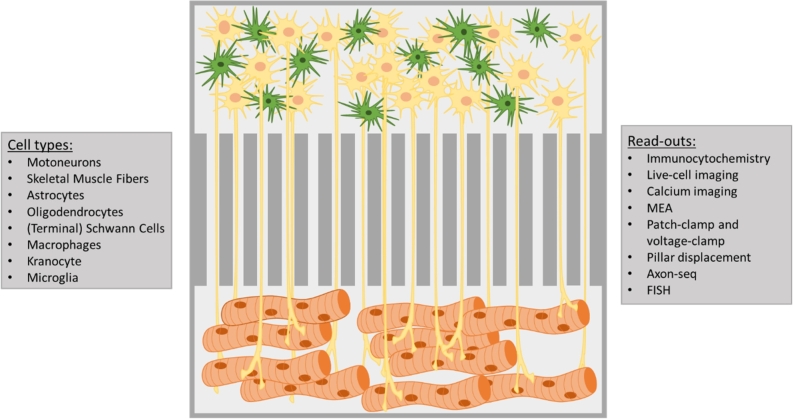
Schematic overview of a simple NMJ-on-a-chip. Motoneurons (yellow) and skeletal muscle cells (brown) are separated by microchannels through which the axons can grow and form neuromuscular junctions. The system can be complemented with additional cell types listed on the left (astrocytes exemplary shown in green). Multiple readouts are possible and dependent on the final design of the platform; possibilities are listed on the right.

Recent years have seen great advances in the development of NMJ-chips and multiple bioengineering approaches have been published [9]. Similar to all organs-on-a-chip, the design is dependent on the desired readout and most systems are custom-made. Generally, a combination of MNs and SkM cells is cultured separately in a compartmentalized chamber, whereby the axons can grow towards the SkM guided through microgrooves/microchannels (Figure 1) to form NMJs. Commonly used cell sources include immortalized cell lines, primary cells, and latterly hiPSC-derived MNs and SkM, with each of them having its advantages and disadvantages [1]. Further, a combination of the different cell sources, i. e., iPSC-derived MNs and primary myoblasts from patients, is used regularly, as well as combining different species [1], [9].

Due to the characteristics of MNs and SkM cells, NMJ-chips offer a variety of readouts (Figure 1). Generally, morphological analyses can be distinguished from functional analyses. For the former, axon growth and formation of NMJs can be followed using time-lapse microscopy as well as immunocytochemistry after fixation of cells in which morphology can be observed in detail. Live-cell imaging can help to elucidate ongoing processes in the axons and the synapses, especially vesicular trafficking/axonal transport and local axonal translation, processes known to be impaired in multiple NMDs [9]. Further, the developmental phenotype of a specific NMD can be investigated. For several NMDs, a so-called “dying back” mechanism has been proposed whereby a dysfunctional NMJ and dying of the axon terminals precedes the loss of the motor neuron [11], which can possibly be observed in these platforms as well. Due to the architecture of the NMJ-chips, the axonal transcriptome can further be analyzed using fluorescence in situ hybridization (FISH, e. g., RNA-scope) or RNA sequencing [12].

Functional analysis of MNs can be achieved by integration of multi-electrode arrays (MEA) into the chip or by a chip design that allows for patch-clamp and voltage-clamp applications. The activity of neurons can moreover be analyzed using calcium imaging. Functional evaluation of the SkM can also be achieved through calcium imaging and MEA recordings or through micropillar displacement, whereby pillars are integrated into the chambers around which the muscle fibers can wrap and whose displacement is recorded upon contraction of the SkM. To evaluate the function of the motor unit itself, MNs are mainly stimulated through methods of optogenetics, electrical or pharmacological stimulation, and the reaction of the SkM is recorded [9].

In addition, new therapeutic compounds can be tested and their effects on motor unit function can be directly evaluated through the functional readouts. The compounds can be screened individually on each component of the NMJ to identify which tissue needs to be treated predominantly and at what time point during development or disease status. Moreover, there are additional cell types known to be important for NMJ development and function that have also been identified to play a role in NMDs. Hence, glia such as terminal Schwann cells, kranocytes, astrocytes, oligodendrocytes, and immune cells (microglia and macrophages) can be integrated into the systems to achieve a better replication of the actual physiological conditions [9], [10]. This is also applicable to the microenvironment of the motor unit, which includes the composition of the extracellular matrix surrounding the NMJ and its impact on the disease.

Although the NMJ-chips seem very versatile, encompassing obvious advantages, all the currently published systems have limitations, discussed below, and further advances are necessary to build more reliable and reproducible systems at larger scales.

## Current limitations and possibilities

The field of organ-on-a-chip technologies is still in its infancy and numerous obstacles must be overcome for these systems to be applied broadly and be accepted as useful alternatives and complements to animal studies, especially in drug testing.

Many current problems arise from the general limitations of working with iPSCs and the 2D and 3D cellular models, for example, the variability of iPSC clones and lines and difficulties to standardize and optimize existing protocols, together with the inter- and intra-batch variability of differentiations, particularly prominent when growing organoids [10], [13]. Additionally, many protocols, such as those to produce spinal cord organoids, were only developed in recent years and in-depth characterization is still ongoing.

These drawbacks are being tackled in various ways. Reprogramming protocols of fibroblasts, lymphocytes, and other cell types have seen huge advancement from using integrating viral systems to footprint-free alternatives [14]. The characterization and evaluation of hiPSC lines has also been improved and more standardized wild-type lines are being distributed worldwide for better comparison between studies. One cell line stands out in particular, namely the WTC-11 line, which is available in multiple modifications and of which whole-genome and transcriptome sequencing analyses are publicly accessible (https://www.allencell.org/cell-catalog.html).

Apart from the variability of the differentiations themselves, the purity of the cell types produced needs to be controlled. This is not only a problem for iPSC-derived cell lines but also for commercially available lines and primary cultures [4]. A possible solution is to use cell sorting before seeding the cells into the chambers.

Modeling human diseases inevitably means that the disease progression needs to be investigated, covering different time points. Especially in NMDs, allelic heterogeneity leads to disease onset at different ages [1]. Unfortunately, current differentiation protocols are not able to produce neurons that go beyond a stage corresponding to that in the second to third trimester [15]. To overcome the problem of maturity, different types of differentiation protocols are available. On one side, there are directed differentiation protocols that try to mimic the physiological maturation by supplying the cells with a variety of growth factors and small molecules similar to what they would experience during development. On the other side, the overexpression of genes that are known to drive the differentiation of specific lineages is often used for accelerated maturation [16]. During the reprogramming of somatic cells, aging hallmarks are mostly lost, which are thought to be relevant for disease development and progression. A useful alternative might thus be the application of so-called transdifferentiation methods, whereby fibroblasts and other cell types are directly differentiated into neurons, skipping the pluripotency stage and keeping their age-related make-up [17], [18]. However, transdifferentiated cells can consequently not be used to study developmental phenotypes. As such, it will become important to use the correct type of differentiation for the disease and the stage/time point of interest. There is always the possibility to use different differentiation protocols to mimic multiple stages of the disease within the same patient. Other solutions to model aging could include the induction of reactive oxygen species, DNA damage, or generating epigenetic modifications related to aging [10].

The organ-on-a-chip field is evolving and research groups all over the world are building and inventing new systems continuously. Currently, these are not standardized and emphasis on different parts of the technologies is put depending on the researchers’ interests. For these systems to become widely accepted and applied for the evaluation of new therapies, international guidelines and standardized readouts will be necessary [19]. Standardization should also include how future systems will be validated and which biologically relevant readouts need to be incorporated into the different chips.

NMJ-chips bear the great possibility to determine the influence of each component of the motor unit on the overall function. This means using a “mix and match” approach whereby healthy muscle cells can be cultured with neurons carrying a pathogenic genetic variant and vice versa. This allows identifying how healthy tissue can modulate its diseased counterparts and which components of the NMJ are primarily and secondarily affected by a certain disorder. It will also help to determine which tissue could be tackled best at what time point to treat a specific disease.

Besides the more general limitations and possibilities of organs-on-a-chip, NMJ-chips and the study of NMDs bears further hindrances. There is now evidence that different types of MNs are affected in different motoneuropathies [9]. To fully evaluate those pathophysiologies, it will be necessary to differentiate cells into these distinct types of MNs, which is not possible to date. Moreover, to evaluate NMJ function, glia that support its formation and function will have to be integrated into the systems. This will make the platforms more complex and the control of each cell (sub)type more challenging. There is also the need to identify media compositions that can support all the integrated cell types. Currently, the lifetime of the cell cultures on organs-on-a-chip is limited. However, proper NMJ maturation requires at least two weeks, and these platforms need to be designed to support the cells for a longer period [20], [21]. Especially for iPSC-derived SkM, achievement of contractility in 2D systems seems to be rare and 3D systems might be needed in the future. Fortunately, a first neuromuscular organoid derived from human embryonic stem cells has been published and shown promising results [22].

## Future hopes

Organ-on-a-chip technologies have the great advantage to be applied in a high-throughput manner. That does not only mean that a multitude of compounds can be tested on one cell line but also that a single compound can be tested on multiple cell lines from different patients simultaneously. This way, the heterogeneity of the population can be recapitulated more reliably, increasing the possibility that a translation into humans will be successful. It also means that responders and non-responders for certain therapies could be identified early on, which bears the chance to eventually generate personalized treatment strategies.

There is also an increasing demand to predict the toxicity and off-target effects of several compounds, like biologicals, oligonucleotides, and small molecules, which could be evaluated with organ-on-a-chip platforms at a large scale. Moreover, clinically relevant biomarkers are still needed for many disorders, including NMDs, and organs-on-a-chip represent a suitable platform for identification and validation of those.

To fully recapitulate a disease and evaluate therapeutic outcomes, ideally, a “body-on-a-chip”/“human-on-a-chip” system has to be created. First approaches are now linking multiple organ-on-a-chip platforms together through microfluidic devices [4]. Therefore, universal media will serve as blood alternatives and systems will need vasculature-like additions. For NMDs, this will also require modeling the blood–brain barrier and blood–spinal cord barrier to determine compounds that can pass through these barriers and also their role in disease pathology. Recently, co-cultures of endothelial cells with MN spheres were established to create a vascularized system [23]. Likewise, artificial spinal fluid will be needed instead of a blood replacement. The field is still far away from generating a “human-on-a-chip.” However, smaller steps towards multi-organ chips are being taken. Regarding NMDs, this for example means to include the motor cortex into the disease evaluation. A first proof-of-concept study fused multiple region-specific organoids to generate a 3D cortico-motor assembloid that was able to transmit signals from the motor cortex organoid into the SkM organoid and induce muscle contractions [24].

Our understanding of the composition of organs and tissues and their interplay is still incomplete, providing a challenge as well as a chance for the organ-on-a-chip field. Currently, the best approach is to try and reverse-engineer human tissues via the identification of key components and characteristics of each organ. This way, organs-on-a-chip can be built step by step with increasing complexity and with the advantage of evaluating the impact of every cell type on the organ’s function in general and disease pathology specifically.

## Conclusion

While there is still a lot of research to be done to generate fully functional NMJ-chips incorporating all necessary cell types and identifying the best readouts, innovative ideas are being brought to life daily. Thus, NMJ-chips are expected to vastly improve our understanding of NMDs.

Although organs-on-a-chip cannot replace animal studies in the respect of analyzing the whole organism, in particular behavior, they can however extend the number of disorders and genetic variants studied, reducing the number of animals needed for scientific research. With the possibility to use NMJ-chips to study NMDs in a high-throughput manner, chances are that drug testing will be accelerated and translatability to actual human disease will be improved.

## Glossary

Microfluidics: Bioengineered miniature devices containing channels, tunnels, and chambers through which very small volumes of fluids (media) can flow.
Multi-electrode array: Device for extracellular electrophysiological measurements. MEAs contain numerous microelectrodes, for *in vivo* or *in vitro* use, that can translate the ion currents generated through neuronal and muscle cell activity into electronic currents detected and measured by the system.
Optogenetics: Method to control neuronal activity with light in cells that have been engineered to express light-sensitive ion channels. Activity can thereby be modulated *in vitro* and *in vivo*.
Organoid: Three-dimensional, miniaturized version of an organ or tissue grown *in vitro* and derived from mainly stem or tumor cells. Multiple (different) organoids or tissues fused are termed assembloids.
Organ-on-a-chip: Miniaturized platform to grow artificial organs that is designed to create a physiological function and response of the system and allows multiple readouts.
